# Supplementary material for: DAU-Net: Dual attention-aided U-Net for segmenting tumor in breast ultrasound images
Source: PLoS One. 2024 May 31;19(5):e0303670. doi: 10.1371/journal.pone.0303670 (PMC11142567; doi:10.1371/journal.pone.0303670)
Supplement: Attachment — Submitted filename: Response sheet_PONE-D-23-35838.pdf [file pone.0303670.s001.pdf]

Manuscript ID: PONE-D-23-35838

We are very grateful to the Editor for considering our manuscript for review and we are very thankful to the reviewers for providing us with valuable suggestions and giving feedback about our manuscript. We have made the required changes to address the editor's and reviewers' comments. Please note that the changes are highlighted in the revised manuscript and herein as blue for Reviewer #1 and green for Reviewer #2 for the convenience of the reviewers. Replies to each of the reviewers' comments are also listed below.

—---

Additional Editor Comments:
The reviewers suggest improvements in several key areas: expanding dataset validation for better generalizability, enhancing language clarity, providing more detailed descriptions of innovations like PCBAM and SWA, clarifying statistical methods and results, and updating the literature review to include the latest research. Additionally, the manuscript should address potential overfitting as indicated in Fig. 4, present comparative predictive results, apply k-fold cross-validation for fairness, and consider the implications of image resizing dimensions. These revisions are essential for enhancing the manuscript's overall quality and relevance to the field.

Response: We are grateful for the constructive feedback from the reviewers and recognize the importance of their suggestions for refining our manuscript. In response to their comments, we have undertaken thorough revisions to address all the feedback provided. We believe that these modifications will greatly improve the overall quality and relevance of the manuscript within the field.

—---

Reviewer's Comments:
Reviewer #1:

Comment #1: The proposed method is only verified on one kind of dataset, which I think is very insufficient. I hope the authors can experiment with other datasets.

Response: We are thankful to the reviewer for his/her suggestions. Please note that we have incorporated the experimental results obtained from an additional dataset, namely the UDIAT dataset, under the section **Experimentation on the UDIAT dataset.**

Comment #2: The English expression of this paper is not very standardized, which brings some confusion to the reader's understanding. I think it could be polished by someone more professional.

Response: We thank the reviewer for this suggestion. Please note that we have improved the English expression in the revised manuscript.

Comment #3: The proposed PCBAM and SWA are the main innovations of this paper. Here, PCBAM is already a relatively conventional method, so it can be considered meaningful. However, the SWA module authors did not describe it accurately enough in to "Shifted Window Attention" section. I did not understand the specifics of the module. Moreover, the authors can refer to these papers: https://doi.org/10.1016/j.media.2023.102980, https://doi.org/10.1016/j.compmedimag.2022.102054, etc.

Response: We appreciate the reviewer's feedback on this. Please note that we have revised and highlighted the section **Shifted Window Attention** in our revised manuscript where we have explained the working of the **SWA** module.

Comment #4: What does Fig. 4 mean? Prove that the model is overfitted. If the results on the validation set are so volatile, are the results of the test set still reliable?

Response: We thank the reviewer for these queries. Please note that we have re-examined our model with fewer iterations and shown the revised results in **Fig 4.**

Comment #5: Table 2 shows the "Mann-Whitney U test" or "p-value test". I think these are two different evaluation methods. Moreover, are all the p-values of the 4 ablation methods shown in the table and the proposed DAU-Net model 0.0286? I remain skeptical.

Response: We appreciate the reviewer's concern on this. Please note that we have obtained the results using Mann-Whitneyu function imported from the scipy.stats package. Also, the obtained results are explained and highlighted under the section, called **Statistical Analysis,** in the revised manuscript.

Comment #6: Is there a difference between "mlp" in formula (1) and "DL" in formula (2)?

Response: We thank the reviewer for this question. "mlp": multi-layered perceptron consists of two successive dense layers (DL). Please note that we have added this information in the revised manuscript.

Reviewer #2:

Breast lesion segmentation is a very interesting work. In this paper, they present a novel Dual Attention-aided U-Net (DAU-Net) for breast ultrasound segmentation. Although the study is interesting, this paper has a few concerns for publication.

Comment #1: In Abstract, it would have been interesting to quantify the results obtained at this level, to guide readers on the performance of your model.

Response: We thank the reviewer for this useful suggestion. Please note that we have mentioned the obtained results in the **Abstract** of the revised manuscript.

Comment #2: The authors have omitted much of the latest research work. Especially the work of 2022 and 2023 is missing. It is recommended that the authors add the latest literature to help readers gain more comprehensive knowledge. The following work can be referred:
[1] https://doi.org/10.1109/TMI.2022.3226268
[2] https://doi.org/10.1016/j.patcog.2023.109728
[3] https://doi.org/10.1016/j.cmpb.2022.107086

Response: We thank the reviewer for this suggestion. It is to be noted that we have added the recommended papers in our revised manuscript.

Comment #3: In Fig. 6, the author needs to display the prediction results of each method

Response: We thank the reviewer for this useful suggestion. Please note that we have included the prediction results of our proposed model in detail including the heatmap segmentation mask additionally in **Fig 5.**

Comment #4: To ensure the fairness of the experiment, k-fold cross validation is necessary. It can be referred to in future research.

Response: We thank the reviewer for this useful suggestion. Please note that we have obtained the experimental results with 5-fold cross validation and included the detailed results under **Table 2.**

Comment #5: Would resizing the original image to 128 be too small?

Response: We appreciate the reviewer's concern on this. Please note that there is no established standard for resized images in the existing literature for this dataset. Different papers have adopted varied image sizes. Therefore, in this paper, we have resized the images to 128x128 to address resource constraints. Resizing to a smaller dimension reduces the computational burden.
