# Supplementary material for: DAU-Net: Dual attention-aided U-Net for segmenting tumor in breast ultrasound images
Source: PLoS One. 2024 May 31;19(5):e0303670. doi: 10.1371/journal.pone.0303670 (PMC11142567; doi:10.1371/journal.pone.0303670)
Supplement: Attachment — Submitted filename: Response to Reviewers DAU_Net_PONE v2.pdf [file pone.0303670.s002.pdf]

Manuscript ID: PONE-D-23-35838R1

We are very grateful to the Editor for considering our manuscript for review and we are very thankful to the reviewers for providing us with valuable suggestions and giving feedback about our manuscript. We have made the required changes to address the editor's and reviewers' comments.

Reviewer #1: Is it true that the training losses and accuracy in Fig. 4 are stable, but the validation losses and accuracy are so volatile? This is a problem that I don't think has been effectively addressed.

Response: We appreciate the reviewer's concern related to Fig. 4. Please find the following observations and experimentation results we have done to address the comment.

1. We conducted an experiment with our proposed model by increasing the size of the input image from 128x128 to 512x512 to assess if resizing the images affects the learning curve. The corresponding curves are shown below.

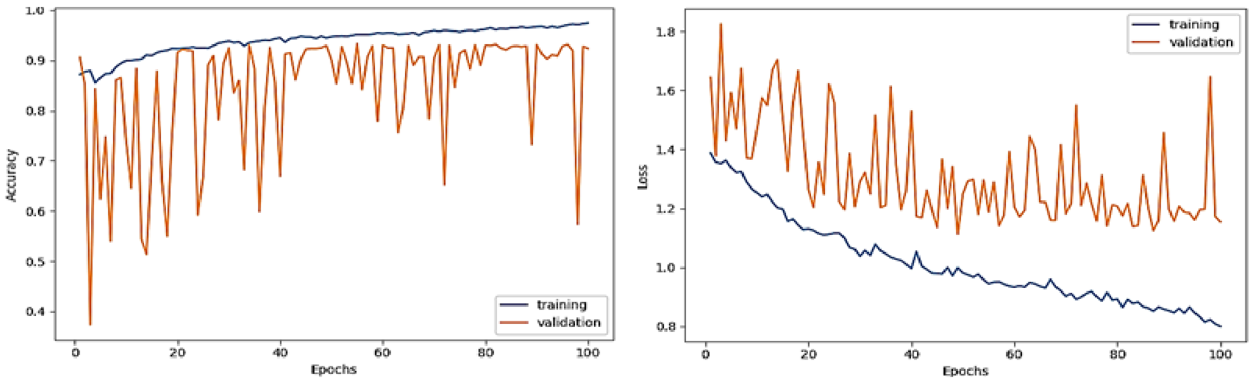

History of accuracy

History of loss

2. Additionally, we conducted another experiment, where we increased the batch size from 4 to 16 to investigate the effect of the batch size on the learning curve during model training. The corresponding curves are displayed below.

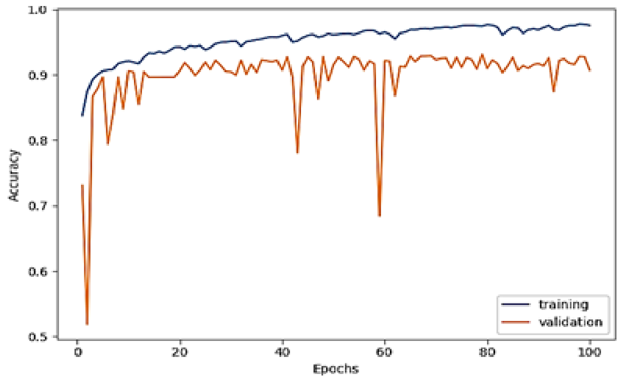

History of accuracy

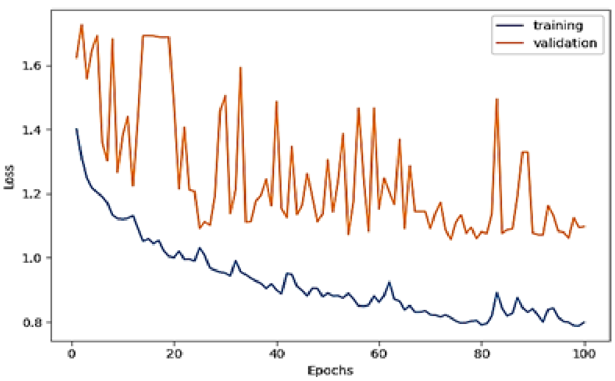

History of loss

3. Moreover, to assess the model's generalizability, we applied data augmentation techniques to the training data (with the setup explained in the paper). The augmentation techniques included dimming or brightening the images, noise addition (gaussian noise). This led to a number of images increased by 4 times. The learning curve during training with data augmentation is shown below.

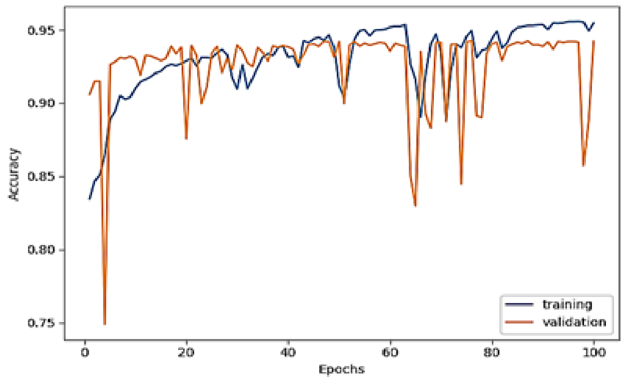

History of accuracy

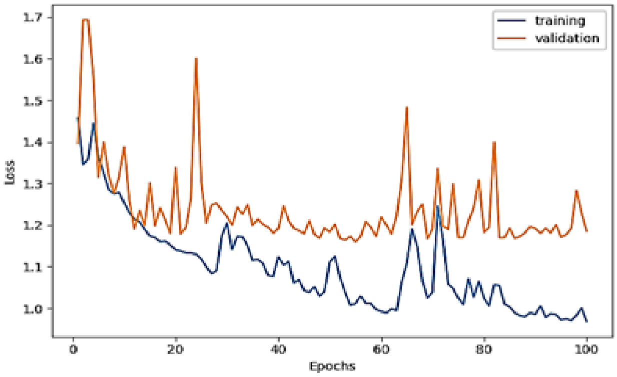

History of loss

4. Furthermore, we extended the number of epochs upto 200 (with the setup explained in the paper) and the corresponding learning curve is depicted below.

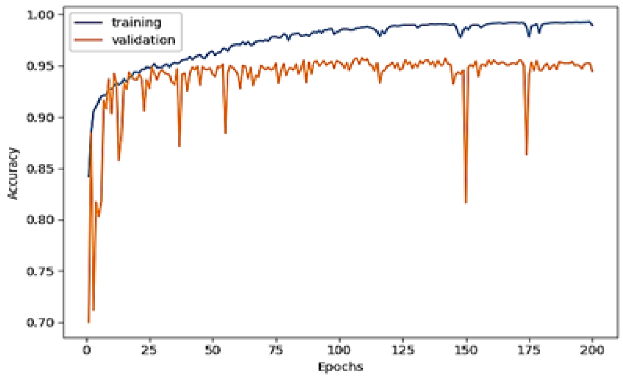

History of accuracy

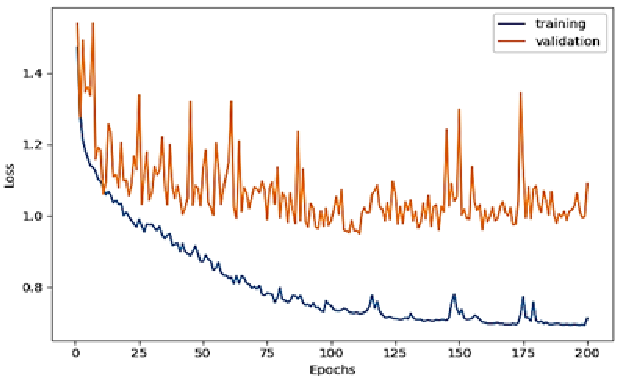

History of loss

Additionally, we have compiled the experiential results discussed above into a table, outlining all the considered metrics. From this analysis, it can be concluded that there is no noticeable change in the metrics with the additional experiments conducted.

Model	Dice	Precision	Recall	IoU
Original	74.27	80.19	75.38	65.97
1.	73.79	79.93	71.24	64.53
2.	71.18	79.85	65.53	63.29
3.	73.51	79.99	72.48	65.71
4.	74.09	81.23	74.52	65.85

From all these above experiments it is clear that the learning curve does not exhibit as much stability in validation metrics as in training metrics in any of the situations. Consequently, we are constrained from including Figure 4 in our revised manuscript.

One potential factor could be the limited diversity within the dataset. To enhance the model's robustness, future research could focus on utilizing larger and more diverse datasets for comprehensive analysis and validation.

Reviewer #2: Thanks for the effort put in as a solution to my doubt. The revised manuscript may be considered for acceptance.

Response: Thank you very much for your feedback and for considering the revised manuscript for acceptance. We greatly appreciate your time and effort in reviewing our work. We are glad that the revisions addressed your concerns and that you found the manuscript suitable for acceptance.
